# Versatile roles of brassinosteroid in plants in the context of its homoeostasis, signaling and crosstalks

**DOI:** 10.3389/fpls.2015.00950

**Published:** 2015-11-04

**Authors:** Shivani Saini, Isha Sharma, Pratap Kumar Pati

**Affiliations:** Department of Biotechnology, Guru Nanak Dev UniversityAmritsar, India

**Keywords:** plant hormones, brassinosteroids, signaling, crosstalk, development

## Abstract

Brassinosteroids (BRs) are a class of steroidal plant hormones that play diverse roles in plant growth and developmental processes. Recently, the easy availability of biological resources, and development of new molecular tools and approaches have provided the required impetus for deeper understanding of the processes involved in BRs biosynthesis, transport, signaling and degradation pathways. From recent studies it is also evident that BRs interact with other phytohormones such as auxin, cytokinin, ethylene, gibberellin, jasmonic acid, abscisic acid, salicylic acid and polyamine in regulating wide range of physiological and developmental processes in plants. The inputs from these studies are now being linked to the versatile roles of BRs. The present review highlights the conceptual development with regard to BR homeostasis, signaling and its crosstalk with other phytohormones. This information will assist in developing predictive models to modulate various useful traits in plants and address current challenges in agriculture.

## Introduction

Brassinosteroids (BRs) are plant specific steroidal hormones, characterized by their polyhydroxylated sterol structure and were first isolated from *Brassica napus* pollen (Grove et al., 1979). BR are regarded as a class of essential plant hormones that plays diverse roles in monitoring broad spectrum of plant growth and developmental processes. They regulate multiple physiological functions including seed germination, cell elongation, cell division, senescence, vascular-differentiation, reproduction, root development, photomorphogenesis, and also respond to various biotic and abiotic stresses (Clouse and Sasse, 1998; Li and Chory, 1999; Sreeramulu et al., 2013; Sharma et al., 2015). Owing to their diverse functions, extensive research has been conducted to promote BR as essential plant growth regulators for modern agriculture (Ikekawa and Zhao, 1991; Divi and Krishna, 2009).

**FIGURE 1:**
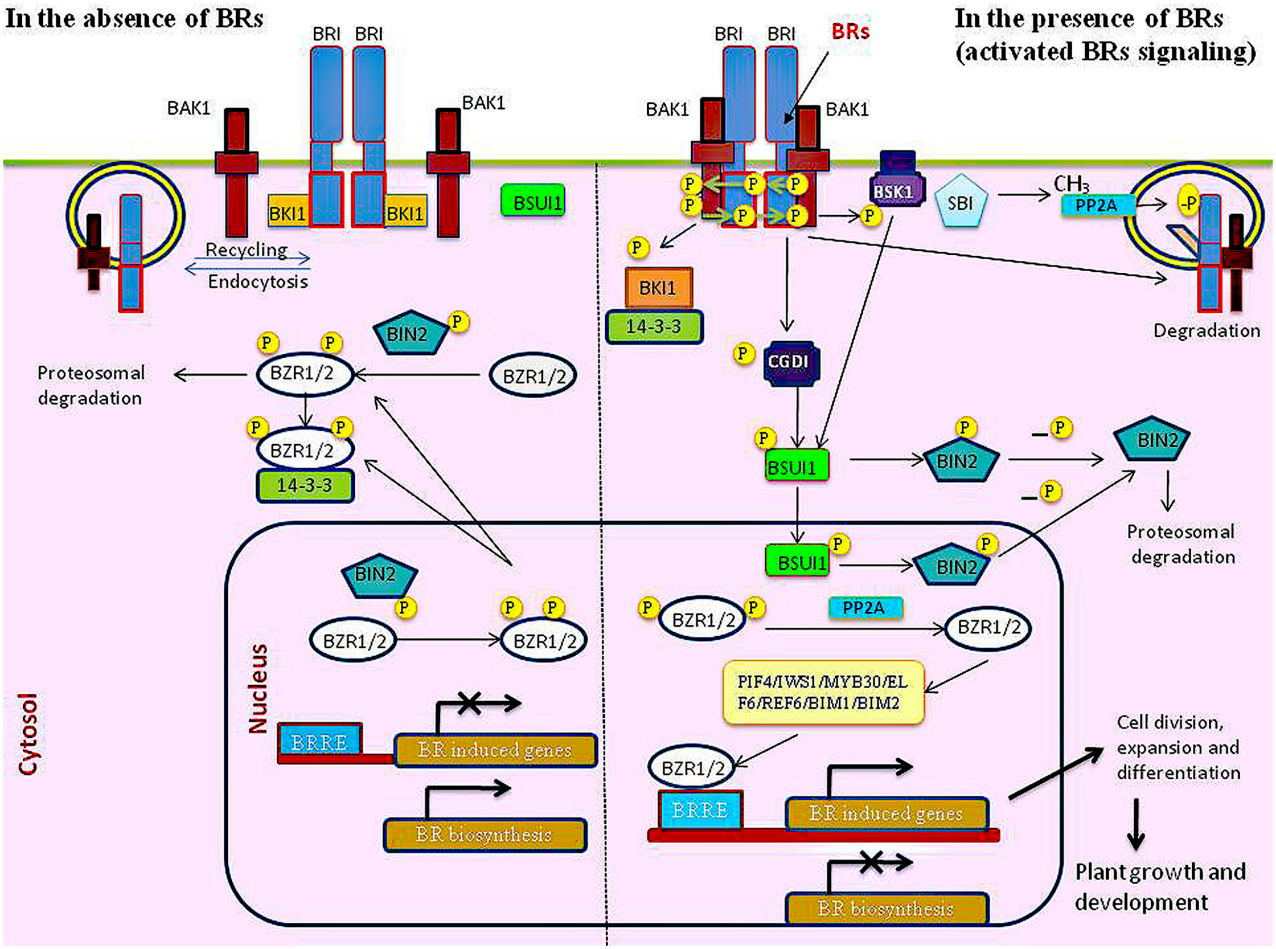
**Model for brassinosteroid (BR) signaling pathway in the absence and in the presence of BR**.

The maintenance and regulation of endogenous level of BR is crucial for various biological functions in plants (Tanaka et al., 2005). BR biosynthesis, transport and degradation are critical components of BR homeostasis and for maintaining the endogenous level of BR in plant. It has been observed that BR-deficient mutants exhibits extreme dwarfism, altered leaf morphology, abnormal vascular development, delayed flowering and senescence, and reduced male fertility (Clouse, 2011). However, excessive application of bioactive BR leads to downregulation of BR-specific biosynthesis genes and an upregulation of BR-inactivation gene, hampering normal development of plants (Bishop and Yokota, 2001; Tanaka et al., 2003, 2005; Zhu et al., 2013a). Moreover, a finely tuned cellular regulation of BR levels is evident from the observation that increase in endogenous BR concentration lead to feedback regulation of the BR metabolic genes, while BR deficient conditions elicit the expression of BR biosynthesis genes to maintain BR homeostasis (Tanaka et al., 2005). Further to understand the BR mediated regulation of several key molecular and physiological functions in plants, extensive research have been conducted over past two decades (Zhu et al., 2013b). BR signaling involves its perception by the cell membrane receptor followed by activation of cascade of phosphorylation events (**Figure 1**) to relay the signal to the downstream partners resulting in the BR-induced gene expression (Belkhadir and Jaillais, 2015). The use of different biological approaches such as mutant screening, microarray, proteomics, protein–protein interaction studies and bioinformatics played vital role in identification and characterization of various components involved in BR signaling (Divi et al., 2010, 2015). Recent studies demonstrate that BR interacts at various level with the signaling components of other phytohormones and regulate process like plant growth and development and stress responses (Hu and Yu, 2014; Tong et al., 2014; Chaiwanon and Wang, 2015; Divi et al., 2015; Yuan et al., 2015). In the above background, the present review focuses on the recent advances in our understanding of the process of BR biosynthesis, transport, degradation and signaling. This information is useful in getting insights into dynamics of BR homeostasis and its implication in modulating various critical functions in plants. Present update also emphasizes the interaction between the key genes and transcription factors of BR with the signaling components of other phytohormones. This information will facilitate in getting insights into a fairly complex process of BR- mediated plant responses.

## Brassinosteroid Homeostasis and its Regulation

BR biosynthesis ensues from intricate network pathways and is mostly modulated by transcriptional regulation of BR biosynthetic genes (Chung and Choe, 2013; Vriet et al., 2013). Various genetic and biochemical studies have elucidated BR biosynthetic pathway which commences with campesterol, a precursor for synthesis of the most active form of BR, brassinolide (BL). Firstly campesterol is converted to campestenal which was initially believed to branch into two parallel pathways, namely the early and late C-6 oxidation pathways involving a chain of reductions, hydroxylations, epimerizations and oxidations which eventually converge at castesterone that leads to the formation of BL (Fujioka et al., 1998). Later studies revealed that BR biosynthetic pathway is a triterpenoid pathway (Choe, 2007; Chung and Choe, 2013). Mevalonic acid serves as a precursor of the triterpenoid pathway and is condensed and transformed to 2,3-oxidosqualene which further undergoes modification to form major plant sterols like sitosterols and campesterols. Depending on the availability of substrate and enzymes, campesterol can be modified by two different enzymes: a C-22 hydroxylase dwarf4/CYP90B1 (DWF4) and a C-3 hydrogenase constitutive photomorphogenesis and dwarf/CYP90A1 (CPD). Since DWF4 can act on multiple biosynthetic intermediates including campesterol and campestenol, the pathway branches to a third early C-22 hydroxylation pathway (Choe et al., 2001; Fujioka and Yokota, 2003). LC-MS and various genetic studies have shown that several other branches are formed by CPD, a C-23 hydroxylase which metabolizes campesterol and other intermediates and has recently been found to participate in a C-3 oxidation as well (Ohnishi et al., 2012). The intermediates formed in above mentioned reactions are further modified and later merge into late C-6 oxidation pathway thus, revealing a certain degree of crosstalk between the parallel pathways manifesting complex networking of BR biosynthesis. Lately, several key genes involved in BR biosynthesis such as *de-etiolated-2 (DET2)*, which is a 5α-reductase, *CPD*, a C-3 oxidase, *DWF4*, a C-22 hydroxylase, *rotundifolia3/CYP90C1 (ROT3)* and *CYP90D1*, C-23 hydroxylases have been characterized to advance the perspective of BR biosynthesis (Wang et al., 2012a; Vriet et al., 2013). Interestingly, monocots like rice and maize, lack the enzyme (CYP85A2) responsible for C-6 oxidation reaction implying that BR synthesis culminates at castesterone in rice (Kim et al., 2008). Recently, it has been shown that in rice, in addition to castesterone, an alternate pathway for biosynthesis of functionally less active C29-BRs exists in order to increase the biological activity of BR in rice. Moreover, C29-BRs even appear to play role in regulation of BR levels by bio-degrading their C-26 demethylated C28-BR analogs to reduce BR activity in planta (Joo et al., 2015).

In contrast to other hormones, BR are not transported to long distances but are rather used in proximity to synthesizing cells. However, they undergo intracellular transport either passively or actively, from their site of synthesis in ER to the plasma membrane where its perception occurs (Symons et al., 2008). Nevertheless, BR are able to exert a long-distance effect by their crosstalk with other hormones like auxins (Symons et al., 2008; Vriet et al., 2013). The short distance transport of BR are suggested to be mediated by some carrier mechanism (BR conjugates formed by binding of BR to fatty acids or glucose) or through specific proteinaceous transporters (Fujioka and Yokota, 2003; Symons et al., 2008). Several proteins belonging to the class of pathogenesis-related (PR) 10 family of proteins, protein family belonging to A or G classes of ABC transporters (ATP binding cassette) and several Sec-14 proteins are potential candidates for mediating BR transport (Markovic-Housley et al., 2003; Deng et al., 2007; Kerr et al., 2011). In the absence of a mode for long distance transport of BR, the spatial and temporal regulation of its homeostasis at the tissue or at the cellular level is extremely crucial for normal growth and development (Symons et al., 2008). BR biosynthesis undergoes two-way regulation mechanism: firstly at the level of endogenous BR by modulating the expression of biosynthetic genes and secondly by inactivating bioactive BR. Most of the BR-specific biosynthesis genes (*DET2, DWF4, CPD, BR6ox1*, and *ROT3*) are feedback regulated (Sun et al., 2010; Yu et al., 2011). Moreover, BR signaling mutant *brassinosteroid insensitive 1* (*bri1*) shows considerable accumulation of endogenous BR as their feedback regulation requires intact BR perception and signaling pathway (Sun et al., 2010). Regulation at the level of transcription is mediated by two major BR signaling transcription factors brassinazole-resistant 1 (BZR1) and BRI1-EMS-suppressor1 (BZR2/BES1) as well as by several other novel transcription factors (CESTA, RAVL1, TCP1) that have been lately identified to regulate the expression of key BR biosynthetic genes such as *CPD* and *DWF4* (Je et al., 2010; Sun et al., 2010; Poppenberger et al., 2011; Guo et al., 2013) Some bHLH like transcription factors like CESTA and TCP are involved in positive regulation of BR signaling (Poppenberger et al., 2011; Wang et al., 2012a; Guo et al., 2013). In rice, another set of transcription factors, ABA insensitive 3/vivaparous1 (ABI3/VP1) and related to ABI3/VP1 (RAV1), not only helps in BR homeostasis by positively regulating biosynthetic genes (*D2, D11* and *BRD1*) but also helps in enhancing the expression of *OsBRI1* (Je et al., 2010).

Brassinosteroid catabolism/metabolism involving various process like acylation, sulphonation, glycosylation etc. play a crucial role in maintaining the optimum levels of bioactive BR in the cell. Novel genes belonging to the family of BAHD acyltransferases, *brassinosteroid inactivator1* (*BIA1)* and *abnormal shoot-1* (*abs-1*) (Roh et al., 2012; Wang et al., 2012a) have been identified to be involved in BR acylation to inactivate BR. Another BAHD acyltransferases pizza (PIZ), have a redundant role with BIA1 in BR inactivation (Schneider et al., 2012). Similarly, bri1-5 enhanced 1 (BEN1) and *Brassica napus* sulfotransferase 3 (BNST3) possessing differential specificities to castesterone and BL inactivate active BR by various mechanism involving reduction and sulfonation (Marsolais et al., 2007; Yuan et al., 2007). A set of glycotransferases enzymes UGT73C6 and its close homolog UGT73C5, catalyze the 23 *O*-glycosylation of CS and BL as part of the inactivation process (Poppenberger et al., 2005). Evidence reveal that these conjugations may serve as temporary storage forms of pool of inactive BR and believed to serve additional functions such as irreversible inactivation, transport, compartmentalization, and protection against cellular removal (Bajguz, 2007; Husar et al., 2011; Piotrowska and Bajguz, 2011). Recent studies have also shown that BR biosynthesis can be regulated by external stimuli like salt and temperature stress (Maharjan and Choe, 2011; Sharma et al., 2013).

## Brassinosteroids Signaling

In the recent past, with the use of various biochemical, genetic and proteomic approaches, a great advancement has been made in our understanding of the BR signaling pathway (Zhu et al., 2013b; Fàbregas and Caño-Delgado, 2014; Belkhadir and Jaillais, 2015). At the plasma membrane (**Figure 1**), BR are perceived by the extracellular domain of the plasma membrane localized leucine rich repeat receptor like kinase (LRR-RLK) BRI1 and its two closely related homologs, BRI1-LIKE 1 (BRL1) and BRI1 LIKE 3 (BRL3), in the nanomolar range (Cano-Delgado et al., 2004). With the recent elucidation of atomic structures of the BRI1 and BRL1 in complex with BL has facilitated in structural understanding of BR perception at the cell surface (Hothorn et al., 2011; She et al., 2011, 2013). BRI1 and BRL1 have similar basic skeleton with 25 and 24 units of LRRs, respectively with a great degree of similarity in overall shape and curvature of the horseshoe-like structure of extracellular domain which is the seat for binding of BR. However, minor structural differences in the BR binding pocket of BRL1 and BRI1 reveals that BR have less affinity to BRI1 as compared to BRL1 as BR binds more strongly to BRL1 as compared to BRI1 (She et al., 2011, 2013). BRL2, which is considered as another BRL1 homolog and responsible for vascular development has also been studied for its ability to bind to BR (Ceserani et al., 2009). It is interesting to note that despite having high sequence identity of BRL2 with BRL1 and possessing conserved Arg588 and Gly690 residues responsible for higher affinity of BRL1 for BL, BRL2 lacks the ability to bind to BR. This could be due to the presence of heavily negatively charged residues located at the inner side of the binding pocket in BRL2 that result in the change in its hydrophobicity to BRs (She et al., 2013).

In the absence of BR (**Figure 1**), BRI1 homodimer remains in an inactive form by the interaction with inhibitory protein BRI1 kinase inhibitor 1 (BKI1) as well as by autoinhibitory function of its cytoplasmic kinase domain (Wang et al., 2005a; Wang and Chory, 2006; Jaillais et al., 2011). Concurrently, active BR insensitive 2 (BIN2), a GSK3/Shaggy-like kinase, phosphorylates BES1/BZR2 family transcription factors and inhibits their activity by protein degradation, reduced DNA binding, and/or cytoplasmic retention by 14–3–3 proteins (Ye et al., 2011; Hao et al., 2013). However, for fully activation of BR signaling, BRI1 heteroligomerise with its co-receptor BRI1-associated receptor kinase1 (BAK1), also known as somatic embryogenesis receptor-like kinase 3 (SERK3) (Li et al., 2002; Nam and Li, 2002). Binding of BR to the 70-amino acids island domain of BRI1 triggers a change in the receptor either in the form of conformational change in the preformed homodimer or receptor dimerisation (Wang et al., 2005a). It results in the autophosphorylation of BRI1 kinase domain activation loop which induces a partial kinase activity of BRI1 leading to the transphosphorylation at Tyr211 residue of BKI1 and resulting in its dissociation from the membrane and thus allowing BRI1 to interact with BAK1 (Wang et al., 2005b; Wang and Chory, 2006). Thus, BR induces the conformational changes in its receptor that are necessary for BRI1-BAK1 interaction (Santiago et al., 2013; Sun et al., 2013). BKI1 also promotes BRI1 signaling by binding to 14–3–3 protein and repressing their negative functions in BR signaling (Wang et al., 2011). BRI1 then interacts with BAK1 to transphosphorylate each other at multiple residues (Wang et al., 2008). Forster resonance energy transfer (FRET) and fluorescence lifetime imaging microscopy (FLIM) approaches have shown that a significant amount of BRI1-BAK1 hetero-oligomers are present at the cell surface even in the absence of BR (Bucherl et al., 2013). It is hypothesized that preassembled BRI1-BAK1 after conjugation with ligand might undergo discrete rearrangements of their respective intracellular kinase domains. This hypothesis derives its significance as similar signaling paradigms is observed in receptor tyrosine kinases in animals (RTKs) which undergo ligand-independent receptor dimerization, followed by dimer reorganization upon ligand binding (Lemmon and Schlessinger, 2010; Bucherl et al., 2013). Activated BRI1 initiates a cascade of phosphorylation events of its downstream plasma membrane bound receptor-like cytoplasmic kinases (RLCKs), BR signaling kinases (BSKs) and constitutive differential growth 1 (CDG1) to transmit the signal from membrane receptors to cytoplasmic regulators of BR signaling (Tang et al., 2008; Kim et al., 2011; Sreeramulu et al., 2013). Mass-spectrometric analysis has shown that BRI1 phosphorylates CGD1 and BSK1 at Ser-234 and Ser-230, respectively and subsequently phosphorylate and activate BRI1-suppressor 1 (BSU1). It has been found that BSU1 can be activated by BRI1 either through BSK1 or CDG1. Furthermore, BSK1 induced activation requires BRI1 kinase activity, whereas CDG1 induced activation does not require BRI1 but is enhanced by BRI1. Thus, either of one set of interacting partners, BSU1-CDG1 or BSUI-BSK1 is the minimum set of components required for transducing the signal from the receptor kinase BRI1 to the GSK3-like kinase BIN2 (Kim et al., 2011). BSU1 then dephosphorylates GSK3 like kinase BR insensitive 2 (BIN2) (Kim and Wang, 2010) to inhibit its function and relieves the inhibitory effect on two master transcription factors of BR signaling, BZR1 and BZR2 also known as BRI1-EMS suppressor1 (BES1) (Wang et al., 2002; Yin et al., 2002, 2005; He et al., 2005). BZR1 and BES1 are then dephosphorylated by protein phosphatase 2A (PP2A) and released from 14–3–3 proteins (Tang et al., 2011), resulting in their nuclear localization to bind to the promoter of their target genes to regulate their gene expression (Sun et al., 2010; Yu et al., 2011; Wang et al., 2012b). Other BRI1 substrates like *Arabidopsis* TGF-*β* receptor-interacting protein-1 (TRIP-1) and transthyretin-like protein (TTL) have also been identified with TTL being putatively linked to inhibition of BRI1 signaling as it binds with higher affinity to kinase-active BRI1 while TRIP-1 being an essential subunit of eIF3 protein translation initiation complex, its phosphorylation by BRI1 is thought to modulate its activity and influence protein translation (Ehsan et al., 2005). Though both BRI1 and BAK1 are classified as serine/threonine kinases, the studies done in past decade has shown that BRI1 has structural features reminiscent of both serine/threonine and tyrosine kinases like insulin receptor, thus providing insights into the evolution of dual-specificity kinases in plants (Wang et al., 2005b; Oh et al., 2009; Macho et al., 2015). Infact, BRI1 possess significant tyrosine kinase activity and it can undergo autophosphorylation on tyrosine residues within the kinase and juxtamembrane domains and can lead to transphosphorylation of Tyr211 in BKI1 as well as tyrosines in other proteins (Oh et al., 2009; Jaillais et al., 2011; Wu et al., 2012). Tyr-831 and Tyr-956 are identified as autophosphorylation sites *in vitro* and *in vivo* with Tyr-956 in kinase subdomain V being essential for activity, while Tyr-831 in the juxtamembrane domain is not essential for kinase activity but plays an important role in BR signaling *in vivo* (Wang et al., 2005b; Oh et al., 2009; Macho et al., 2015).

## Brassinosteroid Regulated Transcriptional Networks

Brassinazole-resistant 1 and BZR2/BES1 are the two major transcription factors of the BR signaling pathway that mediate BR function by regulating the expression of several thousands of genes amounting to about 20% of the genome in *Arabidopsis* (Guo et al., 2013). They share a significant 88% sequence identity at the protein level and a 97% identity in their DNA-binding domain (Wang et al., 2012b). Their structure basically comprises of a bHLH DNA binding domain (DBD), a BIN2 phosphorylation domain containing 22 putative BIN2 phosphorylation sites, a PEST motif (Pro-, Glu-, Ser-, and Thr-rich) involved in protein degradation, and a 14–3–3 binding motif interacting with 14–3–3 upon phosphorylation of BES1 and BZR1 (Tang et al., 2011; Hao et al., 2013). The highly conserved C-terminal domain between two proteins is implicated in the interaction of BZR1 and BES1 with BIN2 (Wang et al., 2002). Though BZR1 and BZR2/BES1 render various similar biochemical and genetically redundant functions, yet the studies conducted on the light grown mutants (*bzr1-1D* and *bes1-D)* show distinctive phenotypes revealing a variation in the level of two proteins either at the level of expression pattern or interaction with other protein partners (He et al., 2005; Sun et al., 2010). With the help of chromatin immunoprecipitation (ChIP) coupled with *Arabidopsis* tiling arrays and promoter element analysis, it is evident that both BZR1 and BES1 have similar DNA-binding specificities. It has been found recently that both BES1 and BZR1 can bind to the BRRE and E-boxes with BRRE primarily enriched in BR-repressed genes and E-boxes are mostly enriched in BR-induced genes (Sun et al., 2010; Yu et al., 2011; Zhu et al., 2013b). Genome-wide protein-DNA interaction analyses as well as the expression profiling have identified about 953 genes targeted by BZR1 downstream from the BRI1-mediated signaling pathway, while BES1 controls 250 genes out of which a small set of 120 genes show an overlap with the BZR1 target genes and thus regulate various genes depending on specific target gene promoter and dimerization partner (Sun et al., 2010; Gudesblat and Russinova, 2011; Yu et al., 2011). Upon activation of BR signaling (**Figure 1**), BES1 and BZR1 bind to their own promoter sequence to induce their expression through a positive feedback loop (Yu et al., 2011). BES1 and BZR1 can regulate or interact with additional transcription factors like AtIWS, BIM and GATA-binding TFs to regulate secondary BR-responsive genes (Sun et al., 2010). BES1 interacts with a bHLH transcription factor BIM1 to synergistically bind to E box sequences present in many BR-induced gene promoters (Yin et al., 2005; Guo et al., 2013). BES1 can also physically interact with interacting-with-spt6 1 (IWS1), which is involved in RNA polymerase II (RNAPII) post-recruitment, transcriptional elongation, RNA export and histone modifications. IWS1 is recruited to target genes by BES1 to enhance gene expression during transcription elongation (Li et al., 2010). BR mediated regulation of gene expression involves epigenetic mechanism also. BES1 directly interacts with a pair of histone demethylases, relative of early flowering6 (REF6) and its homolog ELF6 (early flowering 6) belonging to the class of jumonji domain proteins, to regulate various physiological processes such as flowering time (Noh et al., 2004; Yu et al., 2008). BES1 can also recruit, a histone lysine methyltransferase called set domain group8 (SDG8), which is implicated in histone H3 Lys-36 di- and trimethylation which suggests that epigenetic mechanisms are also involved in regulating the expression of a subset of BR target genes. (Xu et al., 2008; Wang et al., 2014). Topless (TPL), TPL-related proteins (TPRs), and histone deacetylase 19 (HDAC19) were recently found to assemble into a transcriptional repressor module with BES1 (Ryu et al., 2014). Recently, it has been found that BES1 physically interacts to repress transcription factor, brassinosteroids at vascular and organizing center (BRAVO) which acts as a cell-specific repressor of quiescent center divisions in the primary root of *Arabidopsis*. Thus, BES1 modulates quiescent center divisions at the root stem cell niche, by utilizing BRAVO as a regulatory node to control BR-mediated regulation of stem cell quiescence in plants (Vilarrasa-Blasi et al., 2014). Another transcription repressor, myeloblastosis family transcription factor-like 2 (MYBL2) is known to interact with BES1 to down-regulate the expression of BR-repressed genes which is required for optimal BR response (Ye et al., 2012). BR-activated BZR1 have been found to interact with dark and heat-activated transcription factor phytochrome-interacting factor4 (PIF4). They bind to nearly two thousand common target genes, and synergistically regulate these genes, many of which encode transcription factors and proteins that function in the cell wall and chloroplast (Oh et al., 2012). Promotion of cell elongation by BZR1 and PIFs requires activation of the group of a typical bHLH transcription factors, comprising six proteins paclobutrazol resistant (PREs) (Bai et al., 2012; Oh et al., 2012). BZR1 binds to the promoter of the PRE1 gene to enhance its expression while PREs further interact with and inhibit several other HLH/bHLH factors, including ILI1 binding bHLH Protein 1 (IBH1), a negative regulator BR-dependent gene expression (Zhang et al., 2009b). Another representative of the PRE family PRE3, also named activation-tagged BRI1-suppressor1 (ATBS1), is a 93-amino acids atypical bHLH protein, which acts as a positive regulator of BR-dependent gene expression. Since ATBS1 lacks a DNA binding domain, its positive effect on BR signaling results from heterodimerizing and sequestering other bHLH proteins that were negative regulators of BR signaling. It functions by blocking the DNA-binding activity of bHLH transcription factor ATBS1-interacting factor/ILI1–binding bHLH (AIFs/IBH1) that functions as a negative regulator of the BR pathway (Wang et al., 2009). Phytochrome rapidly regulated1 (PAR1) is another atypical HLH proteins lacking proper DNA binding domain. It functions by forming a heterodimer complex with BR regulated PIF4 (PAR-PIF4) and PRE1 (PAR-PRE1) regulating cell elongation and plant development (Hao et al., 2012).

BR regulate various aspects of photomorphogenesis by interacting with other transcription factors like HY5 and GATA. HY5 is a basic leucine zipper transcription factor that functions as a positive regulator of photomorphogenesis and binds to the promoter of over a thousand genes that are also the direct targets of BZR1. Both BZR1 and HY5 regulate the transcription of these genes in opposite ways thereby supporting an antagonistic interaction between BR and light signals (Sun et al., 2010). A novel B-box zinc finger transcritpion factor, BZR1 suppressor1 (BZS1), which functions downstream of both BR and light signaling pathways has been identified to be repressed by BZR1. BZS1 may also interact with Hy5 transcription factor to control expression of a subset of light-responsive genes. Thus, BZS1 is oppositely regulated by light and BR signals to regulate photomorhogenesis responses (Fan et al., 2012). BZR1 represses expression of another positive regulator of photomorphogenesis, GATA2 that controls a subset of genes co-regulated by light and BR. BZR1 directly binds to the promoter of GATA2 to repress its function, while it is post-transcriptionally activated by light signals (Luo et al., 2010). Another connection between light and BR pathways in regulating morphogenesis is provided by two related transcription factors, golden2-like 1 (GLK1) and GLK2. BES1 directly represses the expression of two related transcription factors, (GLK1) and GLK2, that promote the expression of large number of photosynthetic genes and are required for chloroplast development (Waters et al., 2009; Yu et al., 2011) thus, correlating to BR-mediated inhibition of chloroplast development in the dark.

## Interplay of Brassinosteroids with other Phytohormones

Brassinosteroids perform diverse functions due to its interplay with other phytohormones. In response to environmental cues BR interact with different phytohormones such as abscisic acid (ABA), auxin, cytokinin (CK), ethylene, gibberellins (GA), jasmonic acid (JA), polyamines (PA) and salicylic acid (SA), and to regulate myriad aspects of plant growth and developmental processes in plants (Choudhary et al., 2012b; Gruszka, 2013). Recent studies clearly indicate that in response to various intrinsic and extrinsic factors, the signaling components of BR crosstalks with the key genes and transcription factors of other phytohormones and thereby regulates multiple functions in plants. The unraveling of these complicated mechanisms of BR signaling and its collaboration with other molecular networks will be of great importance in improving modern agriculture.

## Brassinosteroid-Abscisic Acid Crosstalk

**FIGURE 2:**
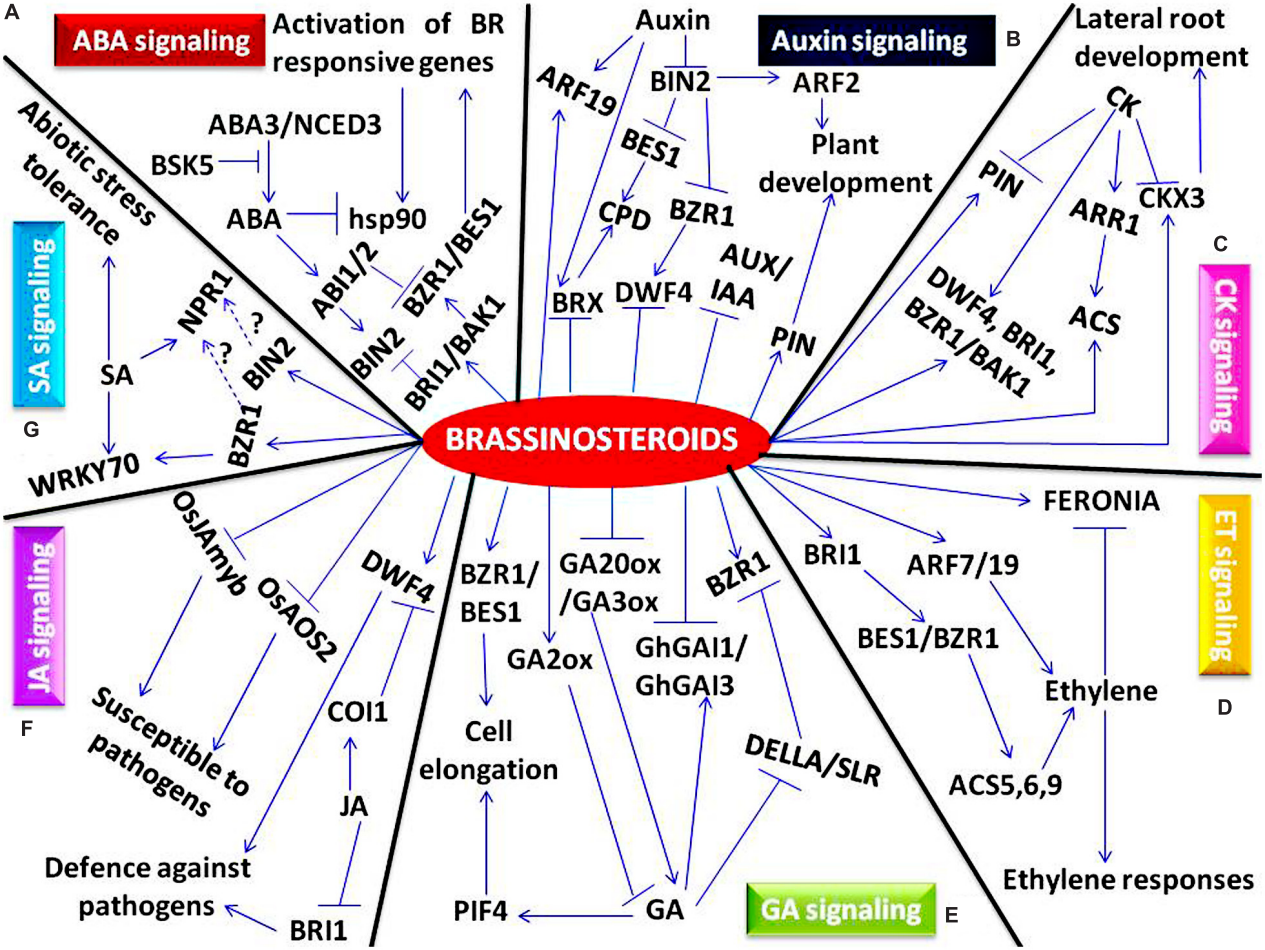
**A schematic model showing crosstalk of various pyhtohormones with brassinosteroids. (A)** Brassinosteroid-abscisic acid (ABA) crosstalk, **(B)** Brassinosteroid-auxin crosstalk, **(C)** Brassinosteroid-cytokinin (CK) crosstalk, **(D)** Brassinosteroid-ethylene (ET) crosstalk, **(E)** Brassinosteroid-gibberellin (GA) crosstalk, **(F)** Brassinosteroid-jasmonic acid (JA) crosstalk, **(G)** Brassinosteroid-salicylic acid (SA) crosstalk. Involvement of different genes and transcription factors as a key component in transcriptional regulation of plant development has been represented. Positive effects are indicated by arrows, bars indicate repression and unknown interactions are represented by dashed arrows.

It is well documented that ABA is required to inhibit seed germination and is also mandatory to establish seed dormancy during embryo maturation. On contrary, BR promotes seed germination indicating the antagonistic interaction between both these hormones (Steber and McCourt, 2001; Finkelstein et al., 2008). Genetic, physiological and biochemical studies have revealed that BR and ABA can co-regulate the expression of 100s of genes (Nemhauser et al., 2006; Zhang et al., 2009a). However, the underlying molecular mechanism and the signaling components involved in this crosstalk are largely unknown. BR and ABA signaling mutants have been analyzed to investigate how ABA inhibits BR signaling (Zhang et al., 2009a). It has been observed that in BR biosynthetic and signaling mutants such as *det2-1* and *bri1*, respectively, the effect of ABA on BR signaling does not rely upon BR perception, but depends on BIN2 (**Figure 2A**), a negative regulator of BR signaling (Zhang et al., 2009a). However, on analyzing ABA signaling mutants, it has been demonstrated that regulatory effect of ABA on BR signaling largely depends upon ABI2 and slightly on ABI1, a PP2C family serine/threonine phosphatases (Zhang et al., 2009a). This study indicates that ABA and BR crosstalks through BR signaling components such as BIN2 and, ABA signaling components such as ABI1 and ABI2. In addition, ABA signaling components act downstream of BRI1 receptor but upstream of BIN2, thus activating BIN2 to negatively regulate BR-mediated responses (Zhang et al., 2009a). Furthermore, BR and ABA have been suggested to play antagonistic roles in regulating seed germination and post-germinative growth processes (Hu and Yu, 2014). ABA inhibits while BR-enhances seed germination and post-germinative growth processes. It has been observed that BIN2, positively regulates ABA responses by physically interacting with ABI5. However, mutations of BIN2 phosphorylation sites on ABI5 made the mutant protein respond to ABA improperly. Hence, the study confirms that BIN2 stabilizes ABI5, by phosphorylating it, thus mediating ABA responses during seed germination. However, BR application inhibits the regulation of ABI5 by BIN2 to antagonize ABA mediated inhibition (Hu and Yu, 2014). Recently, a mutant studies indicate a synergistic correlation between BR and ABA in inducing responses such as, H_2_O_2_ production, *respiratory burst oxidase homolog1* (*RBOH1)* gene expression, NADPH oxidase activity and in mediating heat and oxidative stress tolerance (Zhou et al., 2014). In, ABA biosynthetic mutant, *not*, BR induces transient increase in these responses, however, in BR biosynthetic mutant *dˆ^im^*, ABA induced strong and prolonged increase in these responses. These results indicate that ABA biosynthesis plays a key role in sustaining stress tolerance in BR-induced pathways in plants (Zhou et al., 2014).

It has been demonstrated that high endogenous level of ABA suppresses BR- mediated responses in plants (Divi et al., 2010). In ABA deficient mutant, *aba1-1*, pronounced effects of BR application were observed under heat stress conditions with respect to survival rate due to higher accumulation of heat shock protein90 (HSP90), it indicates that ABA conceals the effect of BR in plant stress responses (Divi et al., 2010). The antagonistic interaction between BR and ABA, has also been established by studying BSK5 protein (Li et al., 2012b). It has been observed that under abiotic stress (salt and drought stress) and upon exogenous phytohormone application (BR and ABA), the transcript levels of *BSK5* gene were enhanced. Moreover, in loss-of-function mutant, *bsk5*, the transcript levels of ABA biosynthesis genes, *ABA3* and *9-cis-epoxycarotenoid dioxygenase* (*NCED3)*, were up-regulated leading to higher ABA levels in *bsk5* plants (**Figure 2A**). It indicates that *BSK5* mutation alters ABA and salt sensitivity and also modifies ABA biosynthetic pathway in plants (Li et al., 2012b).

## Brassinosteroid-Auxin Crosstalk

Crosstalk between BR and auxin regulates myriad aspects of plant growth and developmental processes, (Hao et al., 2013; Saini et al., 2013; Liu et al., 2014; Chaiwanon and Wang, 2015) however, the role of auxin and BR interaction in regulating stress responses has remained elusive (Kissoudis et al., 2014). In *Arabidopsis*, the maintenance of threshold BR level to permit optimum auxin action required for root growth is mediated by *BRAVIS RADIX (BRX)*. It has been demonstrated that the expression of *BRX* gene is strongly induced by auxin while repressed mildly by BR (**Figure 2B**), indicating that *BRX* acts at the nexus of a feedback loop (Mouchel et al., 2006). Interestingly, BR biosynthesis genes *CPD* and *DWF4* are also positively regulated by *BRX* (Tanaka et al., 2005) suggesting a connection between BR biosynthesis and auxin signaling (Mouchel et al., 2006). Furthermore, auxin regulates the expression of BR biosynthesis genes, and hence, are linked directly with BR biosynthesis (Chung et al., 2011). In *Arabidopsis*, the exogenous treatment of auxin dramatically increased the transcript levels of *DWF4* gene leading to enhancement in BR biosynthesis probably through induction of BRX protein (Chung et al., 2011). However, when optimum amount of BR is synthesized, there is a feedback inhibition of *DWF4* gene by BR (**Figure 2B**). The study confirms the antagonistic role of BR and auxin in regulating BR biosynthetic gene, *DWF4* (Maharjan and Choe, 2011; Maharjan et al., 2011). Further, it has also been observed that the transcription of BR biosynthetic genes such as *DWF4* and *CPD*, which catalyzes the rate-determining step and also acts downstream of auxin signaling pathway, are regulated by BZR1 and BES1 (**Figure 2B**), a plant-specific family of transcription factors, respectively (Maharjan et al., 2011). Furthermore, a close relationship between BR and auxin in plant growth and development has been established through interaction between BIN2 and auxin response factors (ARF2). BIN2 mediated phosphorylation of ARF2 results in loss of DNA-binding repression activities of ARF2 (Vert et al., 2008). It leads to the up-regulation of BR regulated genes and also promotes the activity of ARF promoters, enhancing auxin signaling, indicating BR-auxin synergistic interaction. Furthermore, auxin/indole-3-acetic acid (AUX/IAA) proteins which are key players in auxin signaling pathway are also involved in BR responses (**Figure 2B**). Since, AUX/IAA mutants*, iaa7/axr2-1* and *iaa17/axr3-3*, showed aberrant BR sensitivity and aberrant BR induced gene expression due to stabilization of mutants protein caused by failure to interact with SCF^TIR1^ complex, S-phase kinase-associated protein-1 (skp1), such as *Arabidopsis* Skp1-like 1, (ASK1), in plants-the ring box protein 1 (rbx1)-cullin (CUL1)-F box protein, transport inhibitor response1 (TIR1). It indicates that BR and auxin interaction is mediated by SCF^TIR1^ complex and AUX/IAA proteins (Nakamura et al., 2006). Further studies indicate that in rice, auxin treatment enhances the transcript levels of BR receptor gene *OsBRI1* suggesting that auxin regulates the level of BR receptors thus enhancing BR perception. Moreover, the promoter studies of *OsBRI1* gene also indicates that this gene posseses an upstream auxin-response element (AuxRE) motif which is targeted by ARF transcription factors. Further, mutant studies indicate that upon mutation of AuxRE, the induction of *OsBRI1* expression of auxin is abolished and in *arf* mutant the expression of *OsBRI1* gene is down-regulated (Sakamoto et al., 2013). Recently, ChIP and yeast one-hybrid assay demonstrates a link between BR and auxin in controlling lamina inclination which is implicated in plant architecture development and grain yield (Zhang et al., 2014). It has been observed that in *OsGH3.5* and *OsARF19* overexpressing plants, there is reduced content of free IAA at lamina joint resulting in alteration of lamina inclination. Moreover, *OsARF19* also binds to the promoter of *OsBRI1* and positively regulates its expression. In turn, *OsBRI1* activates the expression of *OsBZR1* and its downstream signaling genes to affect lamina inclination (Zhang et al., 2014). The study clearly indicates that *OsARF19* links auxin and BR signaling during regulation of lamina inclination in rice (**Figure 2B**). Furthermore, an antagonistic interaction between BR and auxin has been linked through the transcription factor BZR1 in roots of *Arabidopsis*, to control the spatiotemporal balance of stem cell dynamics required for optimum root growth (Chaiwanon and Wang, 2015). It has been observed that the optimum amount of BZR1 expression patterns required for root growth is established through local BR catabolism, auxin biosynthesis and BR signaling. BZR1 activates the target genes expressed in the transition-elongation zone, but represses genes in the quiescent center and surrounding stem cells, however, on contrary, auxin has an opposite effect to BR on the spatiotemporal gene expression (Chaiwanon and Wang, 2015). BR are also known to play a key role in regulating auxin-responsive genes involved in polar auxin transport such as pinformed3 (PIN3), PIN4 and influx carriers, auxin-resistant1/like aux1 (AUX1/LAXs) (**Figure 2B**) (Hacham et al., 2011, 2012) by affecting their cellular localization. Recently, the actin cytoskeleton was reported to play an essential role in integrating BR and auxin responses. Since, it has been demonstrated that BR alter cytoskeletal configuration in a manner similar to that of auxin. Moreover, BR-mediated reconfiguration of actin cytoskeleton causes delocalization of the PIN2 transporters, thus promoting auxin responses (Lanza et al., 2012). Recent study indicates that in rice, out of 12 *OsPIN* genes, two *OsPINs* (*OsPIN2* and *OsPIN5b*) were induced by drought, heat and cold stresses, however, the other members in rice were suppressed significantly under abiotic stress (Du et al., 2013). Further, it has also been examined that cold stress inhibits the intracellular-cycling of PIN2 and PIN3 resulting in diminished shootward transport of auxin and reduced root’s capability to form auxin gradient required for root growth and patterning (Friml and Palme, 2002; Harrison and Masson, 2008; Shibasaki et al., 2009; Sukumar et al., 2009). Moreover, the effect of BR on auxin transporters has also been studied and it has been observed that BR also represses the expression of several auxin transport related genes, including *PIN3, PIN4, PIN7* and *LAX* gene (Nemhauser et al., 2004). It indicates toward the possible role of BR and auxin crosstalk in abiotic stress tolerance through auxin transporter genes. Furthermore, rice genome acquires seven *YUCCA* genes which encode the rate limiting enzymes for auxin biosynthesis (Yamamoto et al., 2007). It has been observed that all six *OsYUCCA* genes except for *OsYUCCA4* were down-regulated under drought stress. However, under cold stress the transcript levels of *OsYUCCA2*, *OsYUCCA3*, *OsYUCCA6*, and *OsYUCCA7* were strongly upregulated, whereas, heat stress leads to upto five-fold increase in the transcript levels of *OsYUCCA3*, *OsYUCCA6*, and *OsYUCCA7* genes (Du et al., 2013). Further, alteration in the transcript levels of the 40% of BR-upregulated genes has been observed in *yucca* mutants (Nemhauser et al., 2004) indicating BR and auxin crosstalk point. Although the relationship of BR and auxin has been well documented primarily in plant growth and developmental processes, however, further investigations are prerequisite to understand the mechanism of auxin and BR crosstalk involved in abiotic stress tolerance.

## Brassinosteroid-Cytokinin Crosstalk

Cytokinin-brassinosteroid indirectly crosstalks through modulation of auxin transport at the molecular level in regulating lateral root development. BR enhances the expression of auxin eﬄux carriers such as *PIN* genes (**Figure 2C**) which probably aids to maintain local auxin maxima required for root primordium development (Bao et al., 2004). On contrary, CK inhibits the establishment of lateral root primordia and disturbs auxin accumulation by down-regulating the expression of *PIN* genes, indicating an indirect interaction between BR and CK, probably through an unknown mechanism (Benjamins and Scheres, 2008). Furthermore, enhanced root growth due to reduction of CK content was observed in *Arabidopsis* roots upon overexpression of the *cytokinin oxidase/dehydrogenase3* (*CKX3*) gene driven by root-specific promoter PYK10 (Werner et al., 2010). Exogenous application of BR enhances lateral root and leaf length in P10-CKX3 plants under simultaneous effect of CKX3 overexpression and BRI1 ectopic expression (**Figure 2C**). These findings suggest that crosstalk between BR and CKs is involved in the regulation of plant growth and development (Vercruyssen et al., 2011). An evidence for the involvement of BR in regulation of CK level in wheat seedlings has also been provided recently (Yuldashev et al., 2012). CK accumulation, particularly, zeatin derivatives were detected in roots and shoots of 4-day-old wheat seedlings on treatment with BR. However, upon hormone removal, revival of CK concentration to the control level in the roots and shoots was observed, which is suggested to be due to inhibition of the gene encoding CKX protein (Yuldashev et al., 2012). In addition, rice plants overexpressing *isopentyl transferase*, *IPT* gene, resulted in enhanced CK level just before the onset of senescence leading to increase in tolerance to drought stress. The transgenic plants had shown delayed response to stress and exhibited significantly higher grain yield. The observed increase of CKs corresponds with the upregulation of various BR-related biosynthesis genes (*DWF4, DWF5, HYD1)* and BR signaling genes (*BRI1, BZR1*, *BAK1, SERK1*, *BRH1)* (**Figure 2C**). Hence, the crosstalk between BR and CK contribute toward significantly higher grain yield through modification of source–sink relations, thus enhancing drought tolerance (Peleg et al., 2011). Further it has been demonstrated that the CK-mediated inhibition of lateral root initiation does not depend directly on BR level. Since, a report has shown that the *brx-2* mutant, in which BR homoeostasis is hindered, is insensitive to CK-mediated inhibition of lateral root development while, the exogenous application of BR could restore this defect. However, in the presence of CK, the post-embryonic BR treatment could not rescue *brx-2* mutant phenotype (Li et al., 2009) indicating BR-CK independence in the regulation of lateral root development.

Recent studies indicate that in *Chlorella vulgaris*, CK stimulates the accumulation of endogenous BR suggesting the synergistic interaction between BR and CK (Bajguz and Piotrowska-Niczyporuk, 2014). Upon exogenous treatment of 10 nM trans-zeatin (*t*Z) to the *C. vulgaris* culture, there was considerable increase in the level of all endogenous BR by 27–46%. Moreover, the co-application of both BL and trans-Zeatin (*t*Z) lead to highest stimulation in the number of *C. vulgaris* cells and endogenous accumulation of proteins, chlorophylls and monosaccharides, whereas, the lowest was observed upon treatments with 28-homocastasterone (28-homoCS) and 1,3-diphenylurea (DPU) (Bajguz and Piotrowska-Niczyporuk, 2014) indicating BR-CK crosstalk point. Furthermore, BR and CK acts post-transcriptionally to continuously adjust ethylene biosynthesis in response to various environmental factors (Hansen et al., 2009). Crosstalk between CK and BR enhances stability and transcript levels of ethylene biosynthesis gene, ACS. In *Arabidopsis*, CK is perceived by a family of *Arabidopsis* histidine kinase receptors (AHK), which upon autophosphorylation transmit phosphoryl groups to *Arabidopsis* histidine phosphotransfer proteins (AHP). The AHP further activates typeB *Arabidopsis* response regulator (ARR), ARR1, which prevents ACS degradation and activates ethylene biosynthesis (Hansen et al., 2009). Since, it has been observed that *ARR* single mutants, *arr1*, *arr2*, *arr10*, *arr12*, and quadruple mutant *arr 1,2,10,12* showed marked decrease in elevation of ethylene biosynthesis. Hence, the study clearly indicates that CK induction of ethylene biosynthesis is mediated via CK signaling component ARR1. Moreover, BR and CK acts through distinct independent targets but their effect is additive in stabilization of ACS proteins, suggesting BR-CK crosstalk (Hansen et al., 2009). A recent study has ellucidated that BR enhances CK-induced anthocyanin biosynthesis in *Arabidopsis* seedlings (Yuan et al., 2015). Since, it has been observed that mutants defective in BR biosynthesis, *dwf4* and those defective in BR signaling *brassinosteroid insensitive 1-4 (bri1-4)* exhibited delayed expression of the anthocyanin biosynthesis genes and reduced anthocyanin accumulation, as compared to the wild type treated with BR, indicating a positive interaction between BR and CK (Yuan et al., 2015).

## Brassinosteroid–Ethylene Crosstalk

Brassinosteroid and ethylene crosstalk regulate different aspects of plant growth and developmental processes. BR have been identified as a negative regulator of shoot gravitropism, whereas ethylene has been shown to promote gravitropic reorientation in light-grown seedlings (Vandenbussche et al., 2013). It has been suggested that BR and ethylene interact indirectly in regulating shoot gravitropic responses through involving auxin signaling genes (Guo et al., 2008). BR activates *AUX/IAA* (negative regulator of auxin signaling) and inactivates *ARF7* and *ARF19* (positive regulator of auxin signaling), thus inhibiting shoot gravitropic responses. On contrary, ethylene downregulate *AUX/IAA* and enhances *ARF7* and *ARF19* genes to positively regulate shoot gravitropic responses (Vandenbussche et al., 2013). Therefore, ethylene and BR have been found to have an opposite effects on the upward growth of etiolated shoots. Furthermore, ethylene-BR antagonism has also been observed in the case of roots. Ethylene reduces root gravitropic responses (Buer et al., 2006), while BR enhances root gravitropic bending probably by modulating auxin transport (Kim et al., 2007; Vandenbussche et al., 2013). In BR-insensitive mutants, *bri1-301* and *bak1*, delayed root growth and reduced response to the gravitropic stimulus was revealed (Kim et al., 2007). However, in ethylene insensitive mutants, *ein2-5* and *etr1-3* reduced inhibition toward root gravitropic responses was observed (Buer et al., 2006), indicating antagonistic interaction between BR and ethylene in regulating gravitropic responses in plants.

The interaction between BR and ethylene in regulating cell expansion has been consolidated by studying *FERONIA*, which encodes a receptor-like kinase and has a key role in pollen tube reception (Huck et al., 2003; Escobar-Restrepo et al., 2007). Although, *FERONIA* is clearly required for proper fertilization, the knowledge of its role in plant growth and development is scarce. Therefore, mutant studies have been conducted to know the role of *FERONIA* in vegetative plant growth. Further, in *FERONIA* knockdown mutants with reduced *FERONIA* expression, a limited capacity for cell expansion has been observed (Guo et al., 2009). In case of etiolated seedlings, *FERONIA*-dependent BR response subsequently decreases the effect of ethylene on hypocotyl growth. However, loss-of-function of *fer* mutations lead to enhanced ethylene response and reduced BR functions due to change of balance between the positive effects of endogenous BR concentration and inhibitory effects of ethylene on hypocotyl growth. Therefore, the study clearly indicates that *FERONIA* is mandatory for the promotive effects of BR responses in etiolated seedlings (**Figure 2D**) (Deslauriers and Larsen, 2010).

It has been observed that the exogenous application of BR enhanced ethylene biosynthesis in *Arabidopsis* seedlings (Hansen et al., 2009). BR up-regulates the expression of *1-aminocyclopropane-1-carboxylate synthase (ACS)*, the key gene required for ethylene production (Muday et al., 2012). Further, BR acts post-transcriptionally and also increases the stability of ACS proteins such as ACS5, ACS6 and ASC9 (**Figure 2D**) by preventing its ubiquitination mediated by 26S proteasome. Therefore, in response to various endogenous and exogenous signals, *ACS* is regulated by BR to continuously adjust ethylene biosynthesis in various tissues (Hansen et al., 2009).

Furthermore, crosstalk between BR and ethylene through ethylene-inducing xylanase (Eix) plays an important role in plant response to biotic stresses. It has been observed that in specific cultivars of tobacco (*Nicotiana tabacum*) and tomato (*Solanum lycopersicum*) Eix acts as a potential elicitor of plant defense responses. LeEix1 and LeEix2, are the two Eix receptors that belongs to a superclade of leucine-rich repeat receptor-like proteins (RLP). These receptors are able to bind Eix, however, only LeEix2 initiates defense responses. It has been demonstrated that upon application of the Eix elicitor, LeEix1 heterodimerizes with LeEix2 and further, LeEix1 is able to attenuate Eix-induced internalization and signaling of the LeEix2 receptor. Moreover, the activation of attenuating function of LeEix1 requires the binding of LeEix1 to BAK1 and not LeEix2, indicating BR- ethylene crosstalk (Bar et al., 2010). Furthermore, it is also known that BR crosstalks with ethylene to confer abiotic stress tolerance. Since, in another study it has been suggested that ethylene response factor protein (JERF3) activates the expression of oxidative genes, leading to decreased accumulation of ROS and enhanced abiotic stress tolerance (Wu et al., 2008) indicating that ethylene and BR may interact and lead to sequestration of ROS during stress conditions.

The synergistic interaction between ethylene and BR in regulating hyponastic growth has also been demonstrated (Polko et al., 2012). Ethylene is a key regulator of hyponastic growth, which is employed by plants to cope with biotic and abiotic stresses. *ROT3/CYP90C1* encodes an enzyme which mediates C-23 hydroxylation of BR. A mutation in *ROT3* reduces hyponastic growth leading to impairment of local cell expansion and inhibition of BR biosynthesis, indicating that hyponastic growth induced by ethylene is mainly regulated by BR (Polko et al., 2012).

## Brassinosteroid-Gibberellin Crosstalk

Recently, BR are also found to interact with GA to coordinate different physiological processes (Sun et al., 2010; Li et al., 2012a). However, several evidences indicate BR-GA antagonistic interaction in defense related processes against root oomycete *Pythium graminicola*. It has been demonstrated that in several GA-deficient and/or –insensitive mutants, the disease development was more severe. It implies a positive role of GA in providing resistance against *P. graminicola*. Further, it has been demonstrated that susceptibility similar to those observed in BR treated plants was detected when endogenous GA level was disrupted using GA biosynthesis inhibitor, uniconazole (De Vleesschauwer et al., 2012). However, co-application of BR and uniconazole did not cause any additive effect. But treatment of uniconazole along with brassinazole, a BR inhibitor, negated the resistance-inducing effect of brassinazole. It indicates that BR dampens the effective immune response led by GA. Further, it has been demonstrated that the abundance of GA repressors, DELLA and SLR1 is positively regulated by BR. This phenomenon leads to BR mediated suppression of the GA biosynthetic genes such as *GA20ox* and *GA3ox3* induce *GA2ox* expression (**Figure 2E**) which is involved in suppression of GA signaling and its deactivation (De Vleesschauwer et al., 2012). Recently, the crosstalk between BR and GA has been established in regulating plant cell elongation in rice (Tong et al., 2014). It has been suggested that BR promotes GA accumulation by inducing the expression of *D18/GA3ox-2*, one of the GA biosynthetic genes. However, application of exogenous high concentration of BR leads to the activation of *GA2ox-3*, a GA inactivation gene, resulting in inhibition of cell elongation. Moreover, GA inhibits BR signaling as well as its biosynthesis in a feedback inhibiting loop but facilitate cell elongation through activating primary BR signaling pathway upon applying exogenous high GA concentration, indicating BR-GA crosstalk in regulating cell elongation (Tong et al., 2014). The interaction between BR, IAA, and GA on cotton fiber development has been studied in *Gossypium hirsutum* (Hu et al., 2011). A class of DELLA proteins *GhGAII* was down-regulated by BR and auxin treatment during cotton fiber initiation and elongation, suggesting its importance in cotton fiber improvement through genetic modulation of phytohormone strategy. However, the expression of *GhGAI1* and *GhGAI3* were up-regulated by GA during cotton fiber initiation which is an unessential trait for fiber initiation (Hu et al., 2011) indicating BR-GA crosstalk in regulating cotton fiber development. The synergistic interaction between BR and GA leading to cell expansion during photomorphogenesis has been demonstrated through concurrence of BR-activated BZR1 and GA-inactivated DELLA transcription regulators (Gallego-Bartolomé et al., 2012). It has been suggested that GA promotion of cell elongation requires BR signaling, whereas BR or active BZR1 can suppress the GA-deficient dwarf phenotype. DELLA directly interacts with BZR1 and inhibits BZR1-DNA binding both *in vitro* and *in vivo*. This leads to the inhibition in the perception of environmental signals required for regulating cell elongation and seedling etiolation (Bai et al., 2012; Gallego-Bartolomé et al., 2012; Li and He, 2013). Another study also establishes a link between BR and GA, through DELLAs, that acts as a mediator in regulating cell elongation and plant growth. It has been reported that BZR1 interacts *in vitro* and *in vivo* with repressor of ga1-3 (RGA), a member of the DELLA protein family. Mutation studies indicate that in gain-of-function mutant *bzr1-1d*, RGA suppresses root and hypocotyl elongation. The study suggests that ectopic expression of DELLA proteins reduces the abundance and transcriptional activity of BZR1 indicating that BZR1 and RGA antagonizes each other’s transcriptional activity and also acts as a positive and negative regulators of BR and GA signaling, respectively (Li et al., 2012a).

## Brassinosteroid-Jasmonic Acid Crosstalk

The interaction of BR and JA plays crucial roles in plant development, in both biotic and abiotic responses. The F-box protein, coronatine insensitive1 (COI1) is required for JA responses in *Arabidopsis* and plays a key role in JA signaling. The *partially suppressing coi1* (*psc1*) is a leaky mutation of *DWF4*, that encodes a key enzyme in BR biosynthesis leading to inhibition of root growth. Further studies indicate that upon exogenous BR treatment, the partial restoration of JA sensitivity by *psc1* in *coi1-2* background and the JA hypersensitivity of *psc1* in wild-type COI1 background, was eliminated (Ren et al., 2009). In the wild type plants attenuation of JA inhibition of root growth was observed on exogenous BR application. Further, the inhibition of the expression of *DWF4* by JA was found to be dependent on COI1. These results indicate that *DWF4* is located downstream of COI1 and is down-regulated by JA (**Figure 2F**). Therefore, BR antagonizes JA-signaling pathway in root growth and development (Ren et al., 2009). Furthermore, *BAK1* has also been shown to be involved in providing resistance to *Nicotiana attenuata*, against its specialist herbivore, *Manduca sexta* (Yang et al., 2010). The wounding- and herbivory-induced responses were examined on empty vector (EV) and *NaBAK1*-silenced plants generated by virus-induced gene-silencing system. After wounding, *NaBAK1*- silenced plants showed attenuated JA and JA-isoleucine bursts, required for mediating plant defenses against herbivores indicating the critical role of *BAK1* in providing biotic stress resistance against *M. sexta*. EV levels of defensive secondary metabolites, namely, trypsin proteinase inhibitors (TPIs) were observed on simulating herbivory in *NaBAK1*-silenced plants having similar levels of resistance to *M. sexta* larvae. Furthermore, in *NaBAK1*-silenced plants, JA application elicited higher levels of TPI activity than in EV plants. It suggests that given level of JA enhances the accumulation of TPIs on silencing *NaBAK1* (Yang et al., 2010).

It has also been demonstrated that under stress conditions, BR enhances JA level in rice (Kitanaga et al., 2006), which strongly promotes the expression of thionin genes encoding antimicrobial peptides indicating a potential crosstalk point between these two phytohormones. The crosstalk between BR and JA was further studied to understand how these phytohormones interact in the formation of natural defense in tomatoes against insect herbivory. It has been observed that, BR and JA directly affected trichome density and allelochemical content but in a reverse manner (Campos et al., 2009). The defective mutant studies confirmed that JA promotes the traits required for anti-herbivory whereas BR prevented it. Since, the BR-deficient mutant *dumpy* (*dpy)* showed enhanced pubescence, zingiberene biosynthesis, and proteinase inhibitor expression, on contrary, opposite effects were seen in JA-insensitive *jai1-1* mutant, leading to an increased production of defensive traits. Therefore, results from the defective mutants confirmed that JA promotes the traits required for anti-herbivory whereas, BR prevented it. Moreover, it has also been demonstrated that BR acts upstream of the JA signaling pathway, since, *dpy3jai1-1* double mutant showed that *jai1-1* is epistatic to *dpy*. Furthermore, trichome number in *jai1-1* mutants was severely reduced as compared to *dpy*, which presents a high trichome density emphasizing the importance of JA in trichome formation. The study provides BR-JA antagonistic interactions for the defense against herbivory (Campos et al., 2009). Recently, the crosstalk between BR and JA in regulating rice (*Oryza sativa*) innate immunity during infection with the root-knot nematode *Meloidogyne graminicola* has been studied (Nahar et al., 2013). The exogenous application of BR at low concentration (0.1 and 1 μM) induced susceptibility, however, at high BR concentrations (5 and 10 μM), systemic defense against this nematode was observed in the roots (Nahar et al., 2013). It has also been demonstrated that transcript levels of JA biosynthesis gene, *allene oxidase synthase2 (OsAOS2)* (Mei et al., 2006) and JA-induced signaling gene, *OsJAmyb* (Lee et al., 2001) were strongly down-regulated when the BR concentration was low. However, upon high BR concentration, the transcript levels of JA biosynthesis and signaling genes were up-regulated. These results were further validated through exogenous foliar spraying with JA which leads to strong down-regulation of BR biosynthesis and signaling genes, *OsDWF4* and *OsBRI1* (**Figure 2F**), respectively (Nahar et al., 2013), indicating antagonistic interaction between BR and JA in the rice roots. Furthermore, it has been observed that mutants in the BR biosynthesis or signaling pathway accumulate slightly higher amount of the immediate JA-precursor, 12-oxo-phytodienoic acid, and hence consolidating BR and JA pathway antagonism (Nahar et al., 2013).

## Brassinosteroid-Polyamine Crosstalk

Despite the significant progress made over recent years, we are just at the beginning to explore the BR and PA crosstalk. However, the preliminary studies indicate that BR and PA crosstalk is involved in enhancing the ability of stress tolerance potential of plants (Liu and Moriguchi, 2007). Under copper (Cu) stress treatment, the effect of BR on PA tissue concentrations and antioxidant potential of 7-day-old *Raphanus sativus* L. cv. ‘Pusa chetki’ seedlings had been studied (Choudhary et al., 2010). It has been suggested that abiotic and biotic stresses mediate alteration in free PA content (Liu and Moriguchi, 2007). The change in PA level upon BR application might be involved in mitigating the Cu-generated oxidative stress (Choudhary et al., 2010). Furthermore, BR treatment maintains the optimum amount of spermidine concentration required for normal plant growth and specifically increases the production of putrescine required for stress tolerance but decreases the concentration of cadaverine which generates oxidative burst to counteract heavy metal stress (Takahashi and Kakehi, 2010). Further, the co-application of Cu and BR also decreases cadaverine content enhancing superoxide dismutase (SOD) activity required for stress tolerance (Kuznetsov et al., 2009). It indicates the key role of BR-PA interaction in providing abiotic stress tolerance. A recent study provides the effect of exogenously applied BR and spermidine on *R. sativus* plants exposed to lethal concentrations of Cu (Choudhary et al., 2012a). Their interaction affects the expression of genes involved in Cu homeostasis and leads to enhanced tolerance to Cu stress in *R. sativus* (Choudhary et al., 2012a). It has been further suggested that upon co-application of BR and spermidine, the enhanced Cu stress tolerance is met either through the modulation of the expression of genes encoding PA enzymes or the genes that mediates the homeostasis of IAA and ABA (Choudhary et al., 2012a). Therefore, the use of these compounds is prerequisite for sustaining modern agriculture and to unravel the genes and transcription factors involved in BR and PA signaling pathway.

## Brassinosteroid-Salicylic Acid Crosstalk

The potential crosstalk between BR and SA is mediated via *non-expressor of pathogenesis-related genes 1 (NPR1)* and *WRKY70* (**Figure 2G**), encoding a transcription factor working downstream of NPR1 (Divi et al., 2010). In mutant studies, it has been observed that *npr1-1* genotype was thermosensitive, and also showed defects in the expression of PR genes in response to SA (Clarke et al., 2004, 2009; Larkindale et al., 2005). However, in response to epibrassinolide, mere 2.4 fold increase in percent survival of the *npr1-1* seedlings as compared to ninefold increase in wild type was observed (Divi et al., 2010). The study indicates that stress tolerance is mediated by functional NPR1 for manifestation of BR effect, via controlling BR signaling components such as BIN2 and BZR1 (Divi et al., 2010). Further, the existence of the crosstalk between BR and SA plays a pivotal role in response of plants to biotic as well as abiotic stresses. It has been demonstrated that in tobacco as well as in rice, BR acts as an inducer of a broad range of disease resistance. Upon BR treatment, increase in the resistance to the viral pathogen tobacco mosaic virus, the bacterial pathogen *Pseudomonas syringae* pv. tabaci, and the fungal pathogen *Oidium* sp. has been observed in tobacco (Nakashita et al., 2003). However, in rice plants, it has been observed that BR enhances resistance to the fungal pathogen *Magnaporthe grisea* and the bacterial pathogen *Xanthomonas oryzae*. Moreover, it has been suggested that in tobacco, enhancement in the BR mediated resistance does not require SA which has further been consolidated by measuring SA accumulation and its analysis using *NahG* transgenic tobacco (Nakashita et al., 2003). It indicates that BR and SA act independently in providing resistance against pathogens. However, the combined effect of BR and systemic acquired resistance (SAR) inducers provides additive resistance against pathogens. Although, crosstalk may exist between BR and SA signaling pathways for inducing resistance but it is distinct and act independently (Nakashita et al., 2003).

In contrast to the previous view that BR positively regulate plant innate immunity, recent study provides evidence that *Pythium graminicola* exploits BR as virulence factors and takes the hostage of rice BR machinery to cause disease (De Vleesschauwer et al., 2012). Further, it has been suggested that the negative crosstalk between SA and BR pathways leads to immune suppressive effect of BR. Moreover, upon brassinazole treatment, a reduced susceptibility toward *P. graminicola* had been observed in rice plants due to derepression of the master defense regulators of SA pathway such as *NPR1* and *OsWRKY45*. The study indicates that BR-mediated suppression of SA defense responses occur upstream of *NPR1* and *OsWRKY45* but downstream of SA biosynthesis (De Vleesschauwer et al., 2012).

## Conclusion

Brassinosteroids have emerged as a potent phytohormone due to its versatile functions. The wide range of functions is attributed to its multiple targets and complex regulatory mechanisms. Serious and rigorous global efforts are being carried out in understanding the complexity of the mechanism of BR action. Understanding the dynamics of BR homeostasis and unraveling its interactions with other phytohormones will add new dimension to BR research. With the availability of biological resources and introduction of new experimental tools, it is expected that in coming years there will be a significant addition of knowledge in mode of BR action and this could ultimately culminate in ushering a new era in plant developmental and stress biology.

## Conflict of Interest Statement

The authors declare that the research was conducted in the absence of any commercial or financial relationships that could be construed as a potential conflict of interest.
